# Soluble platelet selectin and platelets in COVID-19: a multifaceted connection

**DOI:** 10.1097/MS9.0000000000002302

**Published:** 2024-06-20

**Authors:** Emmanuel Ifeanyi Obeagu, Getrude Uzoma Obeagu, Patrick Maduabuchi Aja, G.I.A. Okoroiwu, N.I. Ubosi, Theophilus Pius, Muhammad Ashiru, Kingsley Akaba, Teddy Charles Adias

**Affiliations:** a Department of Medical Laboratory Science, Kampala International University; b School of Nursing Science, Kampala International University; c Department of Biochemistry, Faculty of Biomedical Sciences, Kampala International University, Ishaka, Uganda; d Department of Biochemistry, Faculty of Sciences, Ebonyi State University, Ebonyi State; e Department of Public Health Science, Faculty of Health Sciences, National Open University of Nigeria, Jabi, Abuja; f Department of Nursing Sciences, Faculty of Allied Health Sciences, Bayero University, Kano, Kano State; g Department of Haematology, University of Calabar, Calabar, Cross-River State; h Department of Haematology and Blood Transfusion Science, Faculty of Medical Laboratory Science, Federal University Otuoke, Bayelsa State, Nigeria

**Keywords:** COVID-19, platelet, soluble platelet selectin

## Abstract

The COVID-19 pandemic has brought to light the intricate relationship between platelets, soluble platelet selectin (sP-selectin), and disease pathogenesis. Platelets, traditionally recognized for their role in hemostasis, have emerged as key contributors to the immunothrombotic complications observed in COVID-19 patients. Concurrently, elevated levels of sP-selectin, indicative of platelet activation and endothelial injury, have been consistently identified in COVID-19 patients and have shown associations with disease severity and adverse outcomes. This multifaceted connection underscores the pivotal role of platelets and sP-selectin in orchestrating thromboinflammation, vascular dysfunction, and disease progression in COVID-19. Platelet activation triggers the release of inflammatory mediators and promotes platelet-leukocyte interactions, amplifying the systemic inflammatory response and exacerbating endothelial injury. Additionally, platelet-derived factors contribute to microvascular thrombosis, further exacerbating tissue damage and organ dysfunction in severe COVID-19. Elevated sP-selectin levels serve as biomarkers for disease severity and prognostication, aiding in risk stratification and early identification of patients at higher risk of adverse outcomes. Therapeutic strategies targeting platelet dysfunction and sP-selectin-mediated pathways hold promise in mitigating thromboinflammation and improving outcomes in COVID-19 patients. Antiplatelet agents, platelet inhibitors, and anti-inflammatory therapies represent potential interventions to attenuate platelet activation, inhibit platelet-leukocyte interactions, and alleviate endothelial dysfunction. A comprehensive understanding of the multifaceted connection between platelets, sP-selectin, and COVID-19 pathogenesis offers opportunities for tailored therapeutic approaches aimed at mitigating thromboinflammation and improving patient outcomes in this complex and challenging clinical setting.

## Introduction

Highlights

- Early predictor of severity: Soluble urokinase plasminogen activator receptor (suPAR) has emerged as a promising biomarker for predicting the severity of COVID-19 progression.
- Coagulation dysfunction marker: suPAR levels correlate with coagulation dysfunction in COVID-19 patients.
- Prognostic indicator: Monitoring suPAR levels provides valuable prognostic information for healthcare providers.
- Dynamic monitoring tool: suPAR levels can be dynamically monitored throughout the course of COVID-19 infection.
- Potential therapeutic target: Targeting suPAR pathways could offer novel therapeutic strategies for mitigating coagulation dysfunction in COVID-19.

The COVID-19 pandemic, caused by the novel coronavirus SARS-CoV-2, has presented a global health crisis with multifaceted challenges. While the respiratory system is a primary target of the virus, emerging evidence suggests that its impact extends far beyond the lungs. One intriguing facet of this expanded pathophysiology is the intricate relationship between soluble platelet selectin (SP-selectin) and platelets in COVID-19^1–5^. SP-selectin, a cell adhesion molecule, and platelets, the small blood cell fragments traditionally known for their role in clot formation, are both integral components of the immune and circulatory systems. However, their involvement in the context of COVID-19 extends well beyond hemostasis. As our understanding of this disease deepens, it becomes increasingly evident that these elements play crucial roles in the inflammatory response, thrombosis, and microvascular dysfunction observed in COVID-19 patients^6,7^.

The COVID-19 pandemic caused by the novel coronavirus SARS-CoV-2 has presented an unprecedented challenge to global public health systems, with diverse clinical manifestations ranging from mild respiratory symptoms to severe pneumonia, acute respiratory distress syndrome (ARDS), and multi-organ dysfunction. While the respiratory manifestations of COVID-19 have been extensively studied, emerging evidence suggests a crucial role for platelets in the pathogenesis of the disease. Platelets, classically known for their hemostatic function in clot formation and wound healing, are increasingly recognized as key players in immune response, inflammation, and thrombotic complications associated with COVID-19. Platelet activation and aggregation have been observed in COVID-19 patients, particularly in severe cases, contributing to the development of thrombosis and vascular complications. This hypercoagulable state is thought to arise from a complex interplay between viral infection, endothelial dysfunction, and dysregulated host immune response. sP-selectin, a biomarker of platelet activation and endothelial injury, has emerged as a potential link between platelets and the pathophysiology of COVID-19. Elevated levels of sP-selectin in COVID-19 patients suggest its involvement in the prothrombotic milieu characteristic of the disease^1–3^.

Endothelial dysfunction, characterized by impaired vascular integrity and increased permeability, is a hallmark feature of COVID-19 pathogenesis. Platelets interact with endothelial cells through various adhesion molecules and signaling pathways, contributing to endothelial activation and dysfunction. sP-selectin, released from activated platelets, may further exacerbate endothelial injury, promoting microvascular thrombosis and organ damage in COVID-19 patients. The dysregulated inflammatory response observed in severe COVID-19 is associated with cytokine storm, immune cell activation, and tissue damage. Platelets play a crucial role in modulating inflammation by releasing pro-inflammatory mediators upon activation. Elevated levels of sP-selectin have been correlated with markers of inflammation in COVID-19 patients, indicating a potential link between platelet activation, inflammatory cascade, and disease severity^4,5^.

## Aim

The aim of this review is to comprehensively explore and elucidate the multifaceted connection between sP-selectin, platelets, and COVID-19. By examining the role of sP-selectin in platelet activation and endothelial dysfunction, as well as its implications for inflammation, viral pathogenesis, and thrombotic complications in COVID-19 patients, this review seeks to provide a deeper understanding of the mechanisms underlying the pathophysiology of the disease. Furthermore, the review aims to discuss the potential therapeutic implications of targeting sP-selectin and platelet activation pathways for mitigating thrombotic complications and improving clinical outcomes in COVID-19. Through a comprehensive analysis of current research findings and emerging insights, this review aims to contribute to the development of effective therapeutic strategies for managing COVID-19-associated thrombosis and vascular complications.

## Rationale

The rationale behind investigating the multifaceted connection between sP-selectin, platelets, and COVID-19 stems from the growing recognition of the significant role played by platelets in the pathogenesis of the disease. While initially considered primarily in hemostasis and thrombosis, platelets are increasingly understood to have intricate interactions with various aspects of the immune response, inflammation, and vascular function. In COVID-19, where thrombotic complications and vascular dysfunction are prominent features, understanding the specific contributions of platelets, particularly through markers like sP-selectin, is crucial for unraveling the disease’s complexities. Furthermore, the exploration of sP-selectin’s involvement in COVID-19 provides insights into potential diagnostic and prognostic biomarkers. Elevated levels of sP-selectin may serve as indicators of platelet activation and endothelial dysfunction, aiding in risk stratification and monitoring disease progression in COVID-19 patients. Additionally, elucidating the mechanisms by which sP-selectin influences inflammation, viral pathogenesis, and thrombotic events can inform the development of targeted therapeutic interventions. Given the urgent need for effective treatments to mitigate thrombotic complications and improve outcomes in severe COVID-19 cases, understanding the role of sP-selectin and platelets offers promising avenues for therapeutic intervention. By targeting platelet activation pathways and modulating sP-selectin levels, it may be possible to attenuate the hypercoagulable state, reduce inflammation-driven tissue damage, and enhance vascular integrity in COVID-19 patients. Thus, investigating the multifaceted connection between sP-selectin, platelets, and COVID-19 holds immense potential for advancing our understanding of the disease pathogenesis and developing novel therapeutic strategies to address its clinical challenges.

## Review methodology

### Search strategy

A comprehensive search was conducted across electronic databases, including PubMed, Scopus, and Web of Science, using relevant keywords such as “COVID-19,” “platelets,” “sP-selectin,” “thrombosis,” “endothelial dysfunction,” and “inflammation.” The search strategy also included manual searching of reference lists from relevant articles and reviews to ensure comprehensive coverage of the literature.

### Inclusion and exclusion criteria

Clear inclusion and exclusion criteria were established to select relevant studies for the review. Inclusion criteria encompassed studies published in peer-reviewed journals, written in English, and focusing on the relationship between platelets, sP-selectin, and COVID-19 pathogenesis. Exclusion criteria included non-English articles, non-peer-reviewed sources, and studies unrelated to the review topic.

### Data extraction and synthesis

Data extraction involved systematically reviewing the selected studies to extract relevant information, including study design, patient characteristics, key findings, and conclusions. Data synthesis was conducted to organize and analyze the extracted information thematically, focusing on key themes such as platelet activation, endothelial dysfunction, inflammation, viral pathogenesis, thrombotic complications, and therapeutic implications.

### Quality assessment

Quality assessment of included studies was conducted to evaluate the methodological rigor and validity of the evidence. Quality assessment tools specific to the study designs (e.g. Cochrane risk of bias tool for randomized controlled trials, Newcastle-Ottawa Scale for observational studies) were utilized to assess the quality of evidence and potential risk of bias.

## SP-selectin in COVID-19

SP-selectin, also known as P-selectin (CD62P), is a cell adhesion molecule that plays a crucial role in the interaction between immune cells, platelets, and the endothelium, especially in the context of inflammation and thrombosis^8^. While COVID-19 primarily affects the respiratory system, emerging research has highlighted the involvement of SP-selectin in the disease’s pathophysiology, particularly in its vascular and systemic manifestations. In COVID-19, the virus can lead to endothelial dysfunction and activation. SP-selectin is expressed on activated endothelial cells and plays a pivotal role in immune cell recruitment to sites of inflammation. This activation of endothelial cells is believed to contribute to the microvascular dysfunction observed in severe COVID-19 cases^9,10^. SP-selectin is expressed on the surface of activated platelets. In COVID-19, there is evidence of platelet activation and the formation of platelet-leukocyte aggregates. This activation can lead to increased thrombosis risk, potentially contributing to the hypercoagulability seen in severe COVID-19^11^. SP-selectin is part of the selectin family of adhesion molecules involved in leukocyte rolling and recruitment at sites of infection or injury. In COVID-19, the virus’s infection of respiratory epithelial cells triggers an immune response and the release of pro-inflammatory cytokines. SP-selectin’s role in mediating leukocyte adhesion to the endothelium contributes to the hyperinflammatory state seen in severe cases, often referred to as a cytokine storm^12^.

COVID-19 is associated with microvascular dysfunction, which can lead to capillary plugging, impaired blood flow, and tissue damage. SP-selectin’s role in promoting platelet-leukocyte interactions and adhesion to the endothelium is believed to contribute to this phenomenon, impacting various organs and potentially exacerbating disease severity^13^. Understanding the role of SP-selectin in COVID-19 has prompted research into potential therapeutic interventions. Targeting SP-selectin or its downstream signaling pathways is being explored as a strategy to mitigate the inflammatory and thrombotic aspects of the disease. Clinical trials investigating the use of anti-inflammatory and antiplatelet agents are ongoing, with the goal of improving patient outcomes^11^. SP-selectin plays a multifaceted role in the pathophysiology of COVID-19, particularly in the context of vascular dysfunction, inflammation, and thrombosis^14^. Further research is needed to fully elucidate the mechanisms involved and to develop targeted therapies that can address these specific aspects of the disease, with the aim of improving the management and treatment of COVID-19.

## Platelets in COVID-19

Platelets, the small cell fragments in our blood, are traditionally known for their role in hemostasis and blood clotting. However, their involvement in COVID-19 has shed light on their broader role in the disease’s pathophysiology^15,16^. COVID-19 is associated with an increased risk of thrombotic events, such as deep vein thrombosis, pulmonary embolism, and microvascular thrombosis. Platelets are integral to the formation of blood clots, and their activation and aggregation play a significant role in the hypercoagulable state seen in COVID-19. Platelet activation can lead to the formation of platelet-rich clots that contribute to these thrombotic complications^17^. Platelets are not only involved in clot formation but also play a role in the body’s immune response. In COVID-19, platelets contribute to the inflammatory response by releasing cytokines, chemokines, and other pro-inflammatory molecules. This can lead to the amplification of the immune response and the phenomenon known as a “cytokine storm,” which is often observed in severe cases^18^. Platelets can interact with endothelial cells, leading to their activation and dysfunction. The SARS-CoV-2 virus can infect endothelial cells, causing a pro-inflammatory and prothrombotic environment. Platelets adhere to these activated endothelial cells, further promoting clot formation and contributing to the microvascular dysfunction observed in COVID-19^19^.

COVID-19 can lead to the formation of platelet-leukocyte aggregates, where platelets adhere to white blood cells (leukocytes). These aggregates can migrate to sites of infection or inflammation, contributing to the immune response. In the context of COVID-19, these aggregates are believed to play a role in both the inflammatory response and thrombosis^20^. Some studies have shown that COVID-19 patients with lower platelet counts on admission may have a worse prognosis. Thrombocytopenia (low platelet count) may be indicative of disease severity. However, the exact mechanisms underlying this association are still under investigation^21^. Understanding the role of platelets in COVID-19 is critical as it sheds light on the systemic and vascular complications of the disease^22^. Researchers are exploring potential therapeutic interventions, including the use of antiplatelet agents and anticoagulants, to manage the hypercoagulable state and reduce thrombotic complications. Further research is needed to fully comprehend the complexities of platelet involvement in COVID-19 and to develop targeted treatments to improve patient outcomes.

## SP-selectin and platelet activation in COVID-19

COVID-19, caused by the novel coronavirus SARS-CoV-2, has presented a myriad of challenges and complexities in our understanding of its pathophysiology^23^. Among the critical aspects of the disease’s progression is the interaction between SP-selectin and platelet activation. This interplay plays a significant role in the hyperinflammatory and prothrombotic state observed in severe COVID-19 cases. SP-selectin, also known as P-selectin (CD62P), is a cell adhesion molecule expressed on the surface of activated platelets and endothelial cells. It facilitates interactions between platelets, leukocytes, and endothelial cells, contributing to immune cell recruitment and the inflammatory response^24^. COVID-19 is associated with platelet activation, leading to increased levels of activated platelets in circulation. Activated platelets play a pivotal role in hemostasis and thrombosis, but in the context of COVID-19, they also contribute to the systemic inflammation seen in severe cases^25^. Platelet activation in COVID-19 can lead to the formation of platelet-leukocyte aggregates, where platelets adhere to white blood cells. These aggregates are believed to migrate to sites of infection and inflammation, contributing to the immune response and promoting inflammation^26^.

The interaction between SP-selectin and activated platelets plays a critical role in the excessive inflammatory response observed in COVID-19, often referred to as a “cytokine storm.” Activated platelets release pro-inflammatory cytokines and chemokines, further amplifying the immune response^8^. Platelet activation is also linked to the prothrombotic state in COVID-19, leading to an increased risk of thrombotic events such as deep vein thrombosis, pulmonary embolism, and microvascular thrombosis^22^. Understanding the interaction between SP-Selectin and platelet activation in COVID-19 has prompted research into potential therapeutic strategies. Clinical trials are exploring the use of antiplatelet agents and anti-inflammatory treatments to manage the prothrombotic and hyperinflammatory aspects of the disease^28^. The interplay between SP-Selectin and platelet activation in COVID-19 is a multifaceted and crucial aspect of the disease’s pathophysiology. It contributes to both the excessive inflammation and the hypercoagulability seen in severe cases. Further research is needed to fully elucidate the mechanisms involved, leading to the development of targeted therapies to mitigate these aspects of COVID-19 and improve patient outcomes^28^.

## The impact on microvascular dysfunction in COVID-19

The COVID-19 pandemic, caused by the SARS-CoV-2 virus, has presented a spectrum of clinical manifestations, including respiratory distress, systemic inflammation, and a unique challenge involving microvascular dysfunction. Microvascular dysfunction refers to impaired blood flow and function of the small blood vessels, including arterioles, capillaries, and venules. In the context of COVID-19, this dysfunction has significant implications for the disease’s pathophysiology^29^: COVID-19 patients often exhibit widespread capillary plugging in various organs, such as the lungs, heart, kidneys, and brain. Microthrombi, composed of platelets and fibrin, can obstruct these tiny vessels, leading to tissue ischemia, which contributes to organ damage^30^. The microvascular dysfunction in the lungs can impair oxygen exchange, leading to hypoxia, which is a common and serious feature of severe COVID-19. Reduced blood flow in the pulmonary microvasculature can lead to ventilation-perfusion mismatches and contribute to respiratory failure^31^. SARS-CoV-2 infection can directly affect endothelial cells, causing their activation and damage. Endothelial dysfunction can disrupt the delicate balance of vasodilation and vasoconstriction, leading to increased vascular permeability and edema^32^.

The presence of activated platelets, leukocytes, and immune cells in the microvasculature contributes to inflammation and further exacerbates microvascular dysfunction. The release of pro-inflammatory cytokines in COVID-19, part of the cytokine storm, can amplify this process^33^. Microvascular dysfunction is not limited to the lungs but can affect various organs. This contributes to the multi-organ involvement seen in severe COVID-19. Organs such as the heart, liver, kidneys, and brain can all suffer from reduced blood flow and ischemic injury^34^. Understanding the role of microvascular dysfunction in COVID-19 has led to investigations into potential therapies. Anticoagulants and anti-inflammatory agents are being explored as strategies to address microvascular complications and improve patient outcomes^35^. Microvascular dysfunction in COVID-19 is a critical aspect of the disease’s pathophysiology, contributing to tissue damage, hypoxia, and multi-organ involvement^36^. The interplay between the virus, endothelial cells, platelets, and the inflammatory response in the microvasculature underscores the complexity of COVID-19. Further research and clinical trials are essential to develop effective interventions that can mitigate microvascular dysfunction and its systemic impact on patients with severe COVID-19.

## Diagnostic and prognostic value

In the context of COVID-19, there is an urgent need for biomarkers that can aid in early diagnosis, risk stratification, and prognostication. sP-selectin has gained attention for its role in reflecting platelet activation and the interplay between thrombosis and inflammation (thromboinflammation). sP-selectin levels rise in response to platelet activation, which is an early event in the pathogenesis of COVID-19. Elevated sP-selectin can therefore serve as an early indicator of disease. Compared to other markers of inflammation and coagulation, sP-selectin provides specific insights into platelet-related pathophysiological changes. This specificity can complement other diagnostic tests, such as D-dimer and C-reactive protein (CRP), to provide a more comprehensive picture of disease activity. Routine measurement of sP-selectin levels in hospitalized patients can aid in identifying those at risk of severe disease progression. Incorporating sP-selectin into existing diagnostic panels alongside other biomarkers can enhance the accuracy of COVID-19 diagnosis and risk stratification^35^.

Elevated sP-selectin levels have been consistently associated with more severe disease manifestations and poorer outcomes in COVID-19 patients. This includes higher rates of ICU admission, need for mechanical ventilation, and mortality. High sP-selectin levels can predict complications such as thromboembolic events, which are common in severe COVID-19. This predictive capability makes sP-selectin a valuable tool for anticipating patient trajectories. sP-selectin can help identify patients who are at higher risk of developing severe complications, allowing for proactive management and tailored therapeutic strategies. Knowledge of a patient’s sP-selectin levels can inform the use of antiplatelet or anticoagulant therapies, potentially reducing the risk of thromboinflammation-related complications. Serial measurements of sP-selectin can monitor disease progression and response to treatment. Declining levels may indicate effective management while rising levels could signal worsening disease or inadequate therapeutic response. Monitoring sP-selectin in the post-acute phase of COVID-19 can help detect long-term complications, such as persistent hypercoagulability, and guide ongoing care. The incorporation of sP-selectin measurements into clinical practice supports a personalized approach to COVID-19 management, tailoring interventions based on individual risk profiles. Accurate risk stratification using sP-selectin can optimize resource allocation in healthcare settings, ensuring that high-risk patients receive appropriate care and monitoring^36^.

## Therapeutic implications of SP-selectin and platelets in COVID-19

The COVID-19 pandemic has revealed a multifaceted relationship between SP-selectin, platelets, and the disease’s pathophysiology^37^. Understanding the therapeutic implications of this connection is crucial for developing strategies to manage and improve patient outcomes. Inhibiting platelet activation is a potential therapeutic strategy to mitigate the hypercoagulable state in COVID-19. Antiplatelet agents, such as aspirin or P2Y12 inhibitors, may be considered to reduce platelet activation and aggregation, lowering the risk of thrombotic complications^38^. Targeting SP-selectin directly or its downstream signaling pathways is being explored as a means to disrupt platelet-leukocyte interactions and reduce the inflammatory response^39^. Research into the development of SP-selectin inhibitors is ongoing, with the aim of reducing the formation of platelet-leukocyte aggregates. Since platelets contribute to the cytokine storm observed in severe COVID-19, anti-inflammatory agents like corticosteroids (e.g. dexamethasone) and interleukin-6 (IL-6) inhibitors (e.g. tocilizumab) have been used to manage the excessive inflammation. Reducing inflammation may indirectly impact platelet activation and improve overall disease outcomes^40^.

Given the hypercoagulable state in COVID-19, anticoagulants such as heparin and low-molecular-weight heparin (LMWH) have been used to prevent and treat thrombotic events. Careful dosing and monitoring are required to balance the anticoagulation benefits with the risk of bleeding^41^. Some treatment protocols involve a combination of antiplatelet agents, anticoagulants, and anti-inflammatory medications to address the various aspects of COVID-19 pathophysiology. These approaches aim to target both the inflammatory and thrombotic components of the disease^42^. Personalized medicine approaches involving patient stratification based on risk factors and biomarkers may help tailor therapies to individual patient needs. Identifying patients at higher risk of thrombosis or severe inflammation may guide treatment decisions^43^. Ongoing clinical trials are investigating the effectiveness of various therapeutic interventions targeting SP-Selectin, platelet activation, and the associated inflammatory and thrombotic pathways in COVID-19. The results of these trials will provide valuable insights into the most effective treatment strategies^44^. The therapeutic implications of SP-Selectin and platelets in COVID-19 revolve around mitigating the hyperinflammatory and prothrombotic aspects of the disease. Various therapeutic approaches, including antiplatelet agents, SP-Selectin inhibitors, anti-inflammatory treatments, and anticoagulants, are being explored to address these complex interactions. Personalized treatment and ongoing clinical trials are essential for optimizing the management of COVID-19 and improving patient outcomes^45^.

## Mechanisms underlying elevated levels of sP-selectin in COVID-19 patients

COVID-19, caused by SARS-CoV-2, has been associated with a wide range of hematological and immunological abnormalities, including elevated levels of sP-selectin. sP-selectin is a marker of platelet and endothelial cell activation, and its elevated levels in COVID-19 patients are indicative of the underlying pathophysiological mechanisms driving the disease’s severity and complications. This discussion explores the mechanisms contributing to the elevated sP-selectin levels in COVID-19, focusing on viral infection-induced endothelial damage, platelet activation, and systemic inflammation^46^.

## Viral infection and endothelial damage

SARS-CoV-2 primarily targets the respiratory system, but it can also infect endothelial cells, which line the interior surface of blood vessels. The virus enters endothelial cells via the angiotensin-converting enzyme 2 (ACE2) receptor, leading to direct cellular injury. This endothelial damage triggers a cascade of events that contribute to the elevation of sP-selectin. SARS-CoV-2 causes direct endothelial damage, resulting in the release of pro-inflammatory cytokines and other mediators that activate the endothelium. Activated endothelial cells express P-selectin on their surface. This expression facilitates leukocyte adhesion and transmigration, promoting inflammation and thrombosis. The activation of endothelial cells leads to the shedding of P-selectin into the bloodstream, increasing sP-selectin levels. The systemic inflammatory response, commonly referred to as the “cytokine storm,” is a hallmark of severe COVID-19. High levels of cytokines such as IL-6, tumor necrosis factor-alpha (TNF-α), and interleukin-1 beta (IL-1β) stimulate endothelial cells, increasing P-selectin expression and shedding. Inflammation-induced vascular permeability allows immune cells and inflammatory mediators to access and activate endothelial cells, perpetuating the cycle of endothelial activation and injury^46^.

## Platelet activation

SARS-CoV-2 can directly interact with platelets, either through ACE2 receptors or other pathways, leading to platelet activation and degranulation. The elevated levels of pro-inflammatory cytokines in COVID-19 stimulate platelet activation indirectly. These cytokines enhance platelet reactivity and aggregation, contributing to the hypercoagulable state observed in COVID-19 patients. Activated platelets express P-selectin on their surface, which mediates interactions with leukocytes and endothelial cells. P-selectin on activated platelets binds to P-selectin glycoprotein ligand-1 (PSGL-1) on leukocytes, forming platelet-leukocyte aggregates. These aggregates further amplify the inflammatory response and promote microvascular thrombosis. Activated platelets adhere to the damaged endothelium, facilitating thrombus formation. The subsequent shedding of P-selectin from these platelets contributes to elevated sP-selectin levels in the bloodstream^48^.

## Role of sP-selectin in thromboinflammation

sP-selectin in the bloodstream can bind to PSGL-1 on circulating leukocytes, promoting their recruitment to sites of endothelial injury and inflammation. By facilitating leukocyte adhesion and migration, sP-selectin plays a central role in the propagation of thromboinflammation, contributing to the severe complications observed in COVID-19 patients, such as microthrombosis and multi-organ failure. The widespread activation of the endothelium and platelets leads to microvascular injury and thrombosis. This is particularly evident in the pulmonary microvasculature, contributing to the severe respiratory complications seen in COVID-19. Microthrombi obstruct blood flow, leading to tissue ischemia and organ dysfunction. Elevated sP-selectin levels reflect this extensive microvascular involvement and serve as a marker of disease severity^46^.

## Therapeutic strategies

The COVID-19 pandemic has highlighted the need for targeted therapies to manage the severe thromboinflammatory complications of the disease. sP-selectin, a marker of platelet and endothelial activation, plays a significant role in these processes. This review examines therapeutic strategies targeting sP-selectin pathways, analyzing ongoing clinical trials, potential side effects, and challenges in targeting these pathways^37^.

## Aspirin

Aspirin inhibits cyclooxygenase-1 (COX-1), reducing thromboxane A2 production and platelet aggregation. Multiple studies are investigating the efficacy of aspirin in reducing thrombotic complications in COVID-19 patients. Gastrointestinal bleeding, hemorrhagic stroke, and allergic reactions are potential risks associated with aspirin use. Balancing the antithrombotic benefits with the risk of bleeding complications is a key challenge in using aspirin^38^.

## P2Y12 inhibitors (Clopidogrel, Ticagrelor)

P2Y12 inhibitors block the P2Y12 receptor on platelets, preventing ADP-mediated platelet activation and aggregation. Trials are ongoing to evaluate the efficacy of clopidogrel and ticagrelor in COVID-19 patients with thromboinflammatory complications. Bleeding, dyspnea (with ticagrelor), and drug interactions are significant concerns. Ensuring patient compliance and managing bleeding risks are critical issues in the clinical use of P2Y12 inhibitors^39^.

## P-selectin inhibitors

### Crizanlizumab

Crizanlizumab is a monoclonal antibody that targets P-selectin, inhibiting its interaction with PSGL-1 and reducing platelet-leukocyte aggregates. Ongoing trials are assessing the effectiveness of crizanlizumab in preventing thromboinflammatory complications in COVID-19. Infusion reactions, potential immunogenicity, and allergic responses are potential side effects. High costs, accessibility, and the need for intravenous administration pose challenges to widespread use^40^.

## Anticoagulant therapies

### Heparin and LMWH

Heparin and LMWH inhibit thrombin and factor Xa, reducing clot formation. Numerous studies are exploring the benefits of heparin and LMWH in preventing and treating thrombotic events in COVID-19. Bleeding, heparin-induced thrombocytopenia (HIT), and osteoporosis with long-term use are concerns. Managing dosing to prevent bleeding while ensuring anticoagulant efficacy is a major challenge^41^.

## Novel therapeutics

### Selinexor

Selinexor is a selective inhibitor of nuclear export (SINE) compound that can modulate inflammation and immune response. Selinexor is being tested for its potential to reduce severe inflammatory responses in COVID-19 patients. Nausea, fatigue, and hematological toxicities are notable side effects. Ensuring efficacy while managing significant side effects requires careful monitoring and patient selection^42^.

## Potential side effects of targeting sP-selectin pathways

### Bleeding risks

Antiplatelet and anticoagulant therapies increase the risk of bleeding, which can range from minor to life-threatening. Regular monitoring of coagulation parameters and patient symptoms is essential to manage bleeding risks effectively^42^.

### Immune reactions

Monoclonal antibodies such as crizanlizumab may cause infusion reactions and immunogenic responses. Pre-medication protocols and close monitoring during infusions can mitigate some of these risks^42^.

### Drug interactions

Polypharmacy in COVID-19 patients, particularly those with comorbidities, increases the risk of adverse drug interactions. Careful consideration of drug interactions and patient history is necessary when prescribing these therapies.

## Challenges associated with targeting sP-selectin pathways

### Clinical implementation

High costs and the need for specialized administration (e.g. intravenous infusions) can limit the accessibility of novel therapies. Adequate healthcare infrastructure is required to support the administration and monitoring of these treatments.

### Patient selection

Identifying patients who will benefit most from targeted therapies while minimizing risks is critical. Continued research to validate sP-selectin and other biomarkers is necessary to refine patient selection criteria.

### Long-term effects

Long-term safety and efficacy data for many of these therapies are still lacking. Ongoing monitoring and research are required to understand the long-term implications of targeting sP-selectin pathways.

Table 1 shows platelet parameters in COVID-19, Table 2 shows sP-selectin Levels in COVID-19, Table 3 shows platelet-leukocyte interactions in COVID-19, Table 4 shows role of platelets in endothelial dysfunction, Table 5 shows diagnostic and prognostic utility of sP-selectin and platelet parameters and Table 6 shows therapeutic interventions targeting platelet dysfunction in COVID-19 (provided by authors).

**Table 1:** Platelet parameters in COVID-19

Parameter	Description
Platelet count	Decreased platelet count observed in severe COVID-19 cases, indicating thrombocytopenia.
Mean platelet volume (MPV)	Increased MPV associated with platelet activation and turnover, reflecting the inflammatory state in COVID-19.
Platelet distribution width (PDW)	Elevated PDW may indicate increased platelet activation and heterogeneity, potentially contributing to thromboinflammation.

**Table 2:** Soluble platelet selectin (sP-selectin) levels in COVID-19

Level	Description
Elevated sP-selectin levels	Increased levels of sP-selectin observed in COVID-19 patients, indicative of platelet activation and endothelial injury.
Correlation with severity	Positive correlation between sP-selectin levels and disease severity in COVID-19, suggesting its potential as a prognostic marker.
Implications for thromboinflammation	sP-selectin implicated in the pathogenesis of thromboinflammation, promoting platelet-leukocyte interactions and endothelial dysfunction

**Table 3:** Platelet-Leukocyte interactions in COVID-19

Interaction	Description
Platelet activation	Platelet activation triggers the release of inflammatory mediators and expression of adhesion molecules, facilitating interaction with leukocytes.
Leukocyte recruitment	Platelet-derived chemokines and adhesion molecules promote the recruitment and activation of leukocytes at sites of inflammation and endothelial injury.
Amplification of inflammation	Platelet-leukocyte interactions amplify the inflammatory response in COVID-19, contributing to systemic inflammation and tissue damage

**Table 4:** Role of platelets in endothelial dysfunction

Role	Description
Endothelial activation	Platelet activation leads to the release of vasoactive substances and cytokines, promoting endothelial activation and dysfunction.
Vascular permeability	Platelet-derived factors contribute to increased vascular permeability, exacerbating tissue edema and inflammation in COVID-19.
Microvascular thrombosis	Platelet adhesion and aggregation on activated endothelium contribute to microvascular thrombosis, a hallmark of severe COVID-19.

**Table 5:** Diagnostic and prognostic utility of sP-selectin and platelet parameters

Utility	Description
Biomarkers for severity	sP-selectin levels and platelet parameters serve as biomarkers for disease severity and prognostication in COVID-19 patients.
Risk stratification	Elevated sP-selectin and altered platelet parameters aid in risk stratification and early identification of patients at higher risk of adverse outcomes.
Monitoring of disease progression	Serial measurements of sP-selectin and platelet parameters enable monitoring of disease progression and response to treatment in COVID-19

sP-selectin, soluble platelet selectin.

**Table 6:** Therapeutic interventions targeting platelet dysfunction in COVID-19

Intervention	Description
Antiplatelet agents	Aspirin and other antiplatelet agents may mitigate platelet activation and thromboinflammation in COVID-19, potentially improving outcomes.
Platelet inhibitors	Targeted inhibitors of platelet activation pathways (e.g. P-selectin inhibitors) could attenuate platelet-leukocyte interactions and endothelial dysfunction.
Anti-inflammatory therapies	Immunomodulatory agents (e.g. corticosteroids, IL-6 inhibitors) may indirectly modulate platelet function by reducing systemic inflammation

IL-6, interleukin-6.

## Conclusion

The COVID-19 pandemic has prompted a deeper exploration of the intricate relationship between SP-selectin, platelets, and the disease’s pathophysiology. As our understanding of these elements in the context of COVID-19 evolves, it becomes evident that they play critical roles in the systemic and vascular complications of the disease. The multifaceted connection between SP-Selectin and platelets in COVID-19 underscores the complexity of the disease’s pathophysiology. These elements play pivotal roles in inflammation, thrombosis, and microvascular dysfunction, contributing to the systemic complications of the virus. As we continue to unravel this intricate web of interactions, we move closer to developing targeted therapies that can address the specific aspects of the disease, ultimately improving the management and treatment of COVID-19 and the outcomes for those affected.

## Ethical approval

Not applicable.

## Consent

Not applicable.

## Source of funding

No funding was received for writing this review paper.

## Author contribution

E.I.O. performed the following roles: conceptualisation, methodology, supervision, draft writing, editing and approval before submission.

## Conflicts of interest disclosure

The authors declare no conflicts of interest.

## Research registration unique identifying number (UIN)

Not applicable.

## Guarantor

Emmanuel Ifeanyi Obeagu.

## Data availability statement

Not applicable.

## Provenance and peer review

Not applicable.
